# Integrated proteomic and genomic analysis to identify predictive biomarkers for valproate response in bipolar disorder: a 6-month follow-up study

**DOI:** 10.1186/s40345-024-00342-x

**Published:** 2024-05-17

**Authors:** Hyunju Lee, Dohyun Han, Kyung Sue Hong, Kyooseob Ha, Hyeyoon Kim, Eun Young Cho, Woojae Myung, Sang Jin Rhee, Jayoun Kim, Tae Hyon Ha, Kang Eun Lee, Hye Won Jung, Yejin Lee, Dongbin Lee, Hyeona Yu, Daseul Lee, Yun Seong Park, Yong Min Ahn, Ji Hyun Baek, Se Hyun Kim

**Affiliations:** 1 Department of Neuropsychiatry, Seoul National University Hospital, 101, Daehak-Ro, Jongno-Gu, Seoul, 03080 Republic of Korea; 2 Proteomics Core Facility, Biomedical Research Institute, Seoul National University Hospital, Seoul, Republic of Korea; 3 Transdisciplinary Department of Medicine & Advanced Technology, Seoul National University Hospital, Seoul, Republic of Korea; 4 Department of Psychiatry, University of British Columbia, Vancouver, BC Canada; 5 Department of Psychiatry, Lions Gate Hospital - Vancouver Coastal Health, British Columbia, Canada; 6 Samsung Institute of Future Medicine, Samsung Medical Center, Seoul, Republic of Korea; 7 Department of Psychiatry, Seoul National University College of Medicine, Seoul, Republic of Korea; 8 Department of Neuropsychiatry, Seoul National University Bundang Hospital, Seongnam, Republic of Korea; 9 Biomedical Research Institute, Seoul National University Hospital, Seoul, Republic of Korea; 10 Medical Research Collaborating Center, Seoul National University Hospital, Seoul, Republic of Korea; 11 Institute of Human Behavioral Medicine, Seoul National University Medical Research Center, Seoul, Republic of Korea; 12 Department of Psychiatry, Samsung Medical Center, Sunkyunkwan University School of Medicine, 115 Irwon-Ro, Gangnam-Gu, Seoul, 03080 Republic of Korea

**Keywords:** Bipolar disorder, Valproate, Genomics, Proteomics, Biomarkers, Precision medicine

## Abstract

**Background:**

Several genetic studies have been undertaken to elucidate the intricate interplay between genetics and drug responses in bipolar disorder (BD). However, there has been notably limited research on biomarkers specifically linked to valproate, with only a few studies investigating integrated proteomic and genomic factors in response to valproate treatment. Therefore, this study aimed to identify biological markers for the therapeutic response to valproate treatment in BD. Patients with BD in remission were assessed only at baseline, whereas those experiencing acute mood episodes were evaluated at three points (baseline, 8 ± 2 weeks, and 6 ± 1 months). The response to valproate treatment was measured using the Alda scale, with individuals scoring an Alda A score ≥ 5 categorized into the acute-valproate responder (acute-VPAR) group. We analyzed 158 peptides (92 proteins) from peripheral blood samples using multiple reaction monitoring mass spectrometry, and proteomic result-guided candidate gene association analyses, with 1,627 single nucleotide variants (SNVs), were performed using the Korean chip.

**Results:**

The markers of 37 peptides (27 protein) showed temporal upregulation, indicating possible association with response to valproate treatment. A total of 58 SNVs in 22 genes and 37 SNVs in 16 genes showed nominally significant associations with the Alda A continuous score and the acute-VPAR group, respectively. No SNVs reached the genome-wide significance threshold; however, three SNVs (rs115788299, rs11563197, and rs117669164) in the secreted phosphoprotein 2 gene reached a gene-based false discovery rate-corrected significance threshold with response to valproate treatment. Significant markers were associated with the pathophysiological processes of bipolar disorders, including the immune response, acute phase reaction, and coagulation cascade. These results suggest that valproate effectively suppresses mechanisms associated with disease progression.

**Conclusions:**

The markers identified in this study could be valuable indicators of the underlying mechanisms associated with response to valproate treatment.

**Supplementary Information:**

The online version contains supplementary material available at 10.1186/s40345-024-00342-x.

## Background

Effective long-term management of bipolar disorder (BD) and its favorable prognosis rely heavily on pharmaceutical therapy due to the chronic nature of the disease. (Judd et al. 2008) However, psychiatrists often encounter challenges in determining appropriate medications because of the heterogeneity of treatment responses. (Samalin and Belzeaux 2023) Although numerous studies have been conducted to explore potential biological markers for drug response prediction in patients with BD (Ho et al. 2020; Sagar and Pattanayak 2017; Perugi et al. 2019), the outcomes are inconclusive.

BD exhibits a high genetic predisposition compared with other psychiatric disorders, with significant genetic heritability. (McGuffin et al. 2003) Over the past two decades, several genetic studies have been conducted to investigate the influence of genomic factors on the outcome of BD drug therapies (Sagar and Pattanayak 2017; Alda and Manchia 2018; Hasler and Wolf 2015; Ziani et al. 2022), and have identified genetic variants associated with drug metabolism, particularly those linked with lithium, neurotransmitter systems, and cellular signaling pathways; however, the predictive value of these variants may vary across studies. (Sagar and Pattanayak 2017; Alda and Manchia 2018; Hasler and Wolf 2015; Ziani et al. 2022) Emerging evidence also suggests the potential use of blood protein biomarkers for treatment response prediction in patients with BD, such as alterations in certain inflammatory markers and complement and coagulation cascades (Perugi et al. 2019; Ziani et al. 2022; Akcan et al. 2018; Gao et al. 2022). Nevertheless, many of these findings await further validation.

The molecular factors influencing drug response in patients with BD include static factors, such as genetic variation, and dynamic factors, such as protein expression. (Fuh et al. 2023) Thus, integrating proteomic and genomic datasets can enhance the prediction of treatment response. (Fuh et al. 2023) Valproate holds a significant role as a medication for BD treatment; however, considerably less research has been conducted on biomarkers specifically associated with valproate, (Zhu et al. 2017) and only a few studies have focused on integrated proteomic and genomic investigations of response to valproate treatment.

The present study aimed to identify biological markers for response to valproate treatment. In order to overcome the limitations of prior studies, we applied a proteomics and genomic approach to identify valid biomarkers. Additionally, we employed methods incorporating both prospective and retrospective measures to evaluate treatment responses.

## Methods

### Ethics

Written informed consent was obtained from all participants before the interviews. The study protocol was approved by the Institutional Review Boards of Seoul National University Hospital (IRB No. 1905-150-1035), Samsung Medical Center (IRB No. 2019-02-038), and Seoul National University Bundang Hospital (IRB No. B-1908-559-404) and adhered to the principles outlined in the Declaration of Helsinki.

### Participants

We recruited individuals who met the criteria for bipolar I or II disorders according to the Diagnostic and Statistical Manual of Mental Disorders 5 (DSM-5) from August 2019 to May 2021 at hospitals affiliated with tertiary care universities in South Korea (Seoul National University Hospital, Samsung Medical Center, and Seoul National University Bundang Hospital). The participants who had received valproate treatment were included in the discovery analysis. The patients received medication prescriptions from the three hospitals, enabling the interviewer to verify the patients’ history of using psychotropic medications. All the participants received standard pharmacological treatment according to the most recent treatment guidelines. (Yatham et al. 2018) Considering the potential differences in treatment responses and prognoses among older individuals with BD, the age range of the participants was limited to 18–60 yrs. (Sajatovic et al. 2015) Patients were categorized into acute and stable groups based on their clinical symptoms during the baseline evaluation. Individuals who met the DSM-5 criteria for manic, hypomanic, or major depressive episodes were assigned to the ‘acute’ group. Patients in the acute group underwent follow-up assessments at baseline (T0), 8 ± 2 weeks (T1), and 6 ± 1 months (T2). The response to valproate treatment was evaluated at T2. The stable group comprised individuals who did not meet the criteria for an acute mood episode and had been receiving pharmacological treatment for a minimum of 2 yrs. The stable group underwent an evaluation, including an assessment of response to valproate treatment, only at baseline. The exclusion criteria for this study were as follows: (1) prior diagnosis of schizophrenia or schizoaffective disorder; (2) history of receiving neuromodulation therapies (such as electroconvulsive therapy, transcranial magnetic stimulation, transcranial direct current stimulation, or deep brain stimulation) within the past month; (3) history of neurosurgery or any central nervous system disease (including epilepsy); 4) severe head trauma resulting in loss of consciousness; (5) history of malignant cancer; (6) history of substance abuse (excluding nicotine or alcohol); and (7) pregnancy or lactation at the time of the initial evaluation. For independent validation analysis, patients with BD in acute status (manic, hypomanic or major depressive episode) who met the above inclusion criteria were recruited from June 2021 to May 2023.

### Clinical assessments

During the baseline evaluation, structured clinical interviews were conducted using either the Structured Clinical Interview for DSM-IV-TR Axis I Disorders or the Mini-International Neuropsychiatric Interview. (First 1997; Hahn et al. 2000) In addition, the DSM-5 criteria were employed to confirm the diagnosis of BD. Participants’ sociodemographic and clinical characteristics, including their medication history, were assessed in the present study. Depressive symptoms were evaluated using the Montgomery–Asburg Depression Rating Scale (K-MADRS) at each evaluation period (T0–T2). (Asberg et al. 1978; Ahn 2005) The state of mania was evaluated using the Young Mania Rating Scale (YMRS), (Young et al. 1978; Jung et al. 2003) and global symptom severity was assessed using the Clinical Global Impression-Bipolar Version (CGI-BP) (Spearing et al. 1997) at each evaluation period (T0–T2). Symptom evaluation and blood sampling were performed simultaneously on the same day.

### Assessment of treatment response using the Alda scale

The Alda scale, initially devised to gauge the response to lithium treatment in patients with bipolar disorders (Scott et al. 2020; Grof et al. 2002), has subsequently shown utility in assessing the treatment response of various medications, including valproate, thus reflecting real-world clinical practice. (Lee et al. 2020; Ahn et al. 2017). Over several years, we have developed a robust evaluation system for our research team. Independent clinicians with a minimum of 3 years of clinical experience carefully reviewed the hospital records and patient responses obtained during the research interviews. Through collaborative discussions, a consensus was reached regarding the treatment response. Furthermore, to ensure consistency among the raters across different hospitals, we conducted regular meetings in which actual patient case vignettes were discussed.

**Fig. 1:**
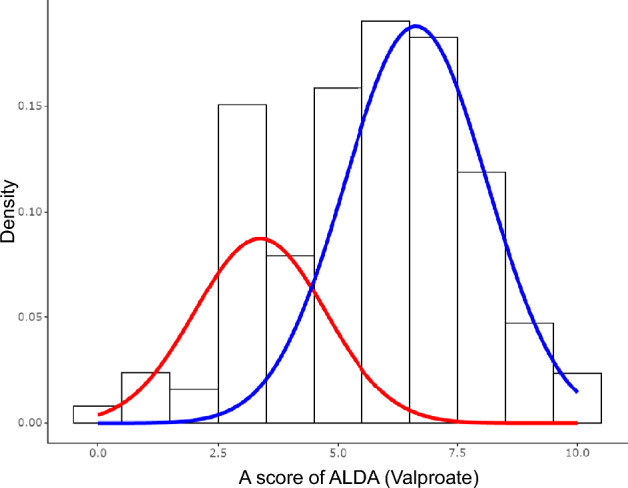
Results of frequentist mixture analysis using Alda A scores (Valproate). This figure shows a histogram of the Alda A scores with two density plots for the two subpopulations. The subpopulations indicate good responders (blue) and moderate/poor responders (red), as assessed using the Alda A scale scores identified utilizing the Bayesian minimum message length method

In the stable group, the clinical records were retrospectively evaluated by clinicians at T0, whereas in the acute group, the scale was assessed by each patient's psychiatrist at T2. The Alda scale comprises two rating sections: the Alda A score, which measures the extent of improvement during treatment on a scale of 0–10, and the Alda B score, which considers confounding factors that independently influence the outcome and includes five items (B1–B5). The overall Alda score was calculated by subtracting the B score from the A score, resulting in a score range of 0–10. (Grof et al. 2002) Higher Alda A and total Alda scores indicated higher response to treatment. The difference in the treatment duration between the acute and stable groups led to a higher Alda B score in the acute group, which contributed to the disparity in the total score. To reduce variations in the treatment duration and vulnerability associated with the Alda B score, (Scott et al. 2020) we used the Alda A score to evaluate treatment response. Furthermore, we categorized patients into two groups based on their response to treatment: responders and non-responders. Using frequentist mixture analysis (Fig. 1), we established the most appropriate theoretical model comprising two components, as indicated by the lowest Akaike Information Criterion (620.08) and Bayesian Information Criterion (626.00) values. A cutoff point was determined as a total score of 5. Therefore, individuals with a score of ≥ 5 were categorized as ‘valproate responders (VPAR),’ whereas those with a score of ≤ 4 were categorized as 'valproate non-responders (VPANR).'

### Plasma protein quantification using multiple reaction monitoring mass spectrometry

For clinical plasma samples, proteolytic digestion was performed with trypsin using a one-step digestion procedure, as previously described. (Lee et al. 2021; Kim et al. 2021; Rhee et al. 2020) Subsequently, the resulting peptides were acidified with 10% trifluoroacetic acid and desalted using homemade C18-StageTips, as previously described. (Han et al. 2014) Mass spectrometry (MS) analysis was performed in the positive ion mode using an Agilent 6495A triple quadrupole instrument; 92 proteins, which included 158 peptides and 474 transitions, were chosen for the targeted multiple reaction monitoring (MRM)-MS. The details of the candidate marker selection process were presented in our previous article (Lee et al. 2024). Supplementary Table S1 presents an overview of these proteins and their associated peptides. The raw data from the MRM-MS analysis were then processed using Skyline v. 20.2 (MacCoss Lab, Seattle, WA, USA), where the peak area values for the transitions were computed. The specific procedures for preparing plasma samples and quantifying proteins using MRM-MS have been previously described. (Lee et al. 2022) The obtained blood samples were divided into two analysis points and analyzed, and the Combat algorithm (http://genepattern.broadinstitute.org) was used to mitigate batch effects. (Reich et al. 2006) Principal component analysis was performed to validate the successful correction of batch effects, with no other factors impacting peptide distribution (Supplementary Figure S1). To account for skewed data distribution, the values underwent a log2 transformation. In addition, the normality of the protein data was assessed using the Shapiro–Wilk test.

### Selection of single nucleotide variants and genotyping

Although the sample size in the present study is limited for a genetic association study, genetic association analyses of candidate genes were performed as a validation modality to confirm the proteomic results. We selected genes that directly encode proteins and were significantly associated with response to valproate treatment. Among the 27 selected genes, we incorporated 1627 single nucleotide variants (SNVs) located within a 50 kb region upstream and downstream of both the exonic and intronic regions of each gene. Genotype data were obtained from the Korea Biobank Array 1.0, commonly known as the Korean Chip (KCHIP, Seoul, Republic of Korea). Genomic DNA was extracted from peripheral blood leukocytes using a Wizard Genomic DNA Purification Kit (Promega, Madison, WI, USA) following the manufacturer’s instructions. Subsequently, SNV genotyping was performed in a commercial laboratory using KCHIP 1.0. The chip used was based on the Axiom™ (Affymetrix, Santa Clara, CA, USA) and developed by the Center for Genome Science at the Korea National Institute of Health. The array comprises > 833,000 markers specifically optimized for the Korean population, encompassing tag SNVs, variants with various frequencies (common and rare), and functional variants derived from the sequencing data of > 2,500 Koreans. To maintain data integrity, we adhered to the quality control procedures outlined in the Korean Biobank Array protocol, which included the evaluation of several parameters to eliminate study samples and variants that did not satisfy the required quality criteria. These parameters encompassed the following criteria: a variant call rate < 0.99, a Hardy–Weinberg equilibrium p-value < 0.000001, minor allele frequency < 0.01, duplicate SNVs, samples exhibiting first- or second-degree relatedness, a sample call rate < 0.95, excessive heterozygosity, sex mismatches, and deviations in the principal component analysis. We also evaluated genetic relatedness using KING. (Manichaikul et al. 2010).

### Statistical analyses

Categorical data were examined using the chi-square test, whereas continuous variables, including proteomic data, were analyzed using analysis of variance with multiple comparisons using Tukey’s honestly significant difference, independent Student’s t-test, and Mann–Whitney U test.

Linear mixed-effects analysis was performed to investigate whether there were distinct trends over time (T0-T2) in clinical symptoms (K-MADRS, YMRS, and CGI-BP-Overall) and the expression of the 158 peptides between the VPAR and VPANR groups. The analysis was adjusted for covariates, including patient age, (Tanaka et al. 2018) sex, (Miike et al. 2010) body mass index (BMI), type of BD (BD-I and BD-II), and mood state (depression vs. (hypo)mania), to account for their influence. Differentially expressed peptides were deemed significant when the p-value between each group was < 0.05. To address the issue of multiple comparisons, p-values adjusted using the Benjamini–Hochberg false discovery rate (FDR) method were also considered.

Association analyses of the candidate genes were performed using the R software. Linear regression analyses were performed with the Alda A score as the dependent variable, whereas logistic regression analyses were performed with valproate responsiveness and non-responsiveness as the dependent variables. Age, sex, and the BD subtype were used as covariates. Post-hoc analyses were performed using the FDR method for each gene.

Bioinformatics analysis was performed using the Database for Annotation, Visualization, and Integrated Discovery (version 2021; (http://david.ncifcrf.gov/). Huang et al. 2009) Cytoscape version 3.8.2 was used for network analysis and visualization. (Shannon et al. 2003) Statistical analyses were performed using the SPSS (version 25.0; BM Inc., Armonk, NY, USA) and R Statistical Software (version 4.3.0; R Core Team, Vienna, Austria; https://www.R-project.org/).

## Results

### Demographic and clinical characteristics

The final analysis of the discovery analysis included 163 participants categorized based on the Alda A score into the following groups: stable-VPAR (n = 69), acute-VPAR (n = 72), and acute-VPANR (n = 22); stable-VPANR (n = 7) was excluded from the analysis due to an insufficient number of samples (Supplementary Figure S2). Table 1 presents the demographic and clinical features of the different groups for comparison. The average age and prevalence of BD-I were higher in the stable group than in the acute group. The acute-VPAR group had a lower BMI than the other groups. The age at BP diagnosis and the frequency of hospitalization were greater in the stable-VPAR group than in the acute-VPAR group. In contrast, the prevalence of quetiapine use was higher in the acute groups than in the stable group. The prevalence of lithium use was higher in the acute-VPANR group than in the other groups, whereas the prevalence of risperidone use was higher in the acute-VPANR group than in the stable-VPAR group (Table 1). The independent validation dataset comprised 50 VPARs and 25 VPANRs. There were no significant differences in the sociodemographic and clinical features between the discovery dataset and the validation dataset except family history of mood disorders (Supplementary Table S2).

**Table 1:** Comparison of sociodemographic and clinical characteristics among stable-valproate responders, acute-valproate responders, and acute-valproate non-responders

	Total	Stable-VPAR (a)	Acute-VPAR (b)	Acute-VPANR (c)	Statistics^†^	*p*-value	post-hoc
	(n = 163)	(n = 69)	(n = 72)	(n = 22)			
Age, year	32.2 ± 10.8	37.8 ± 11.2	28.0 ± 8.5	28.4 ± 8.8	19.8	< 0.001	a > b,c
Sex, female	54 (33.1)	41 (59.4)	51 (70.8)	17 (77.3)	3.3	0.193	
Education, year	14.9 ± 1.9	15.2 ± 2.1	14.8 ± 1.9	14.4 ± 1.5	1.6	0.199	
BMI, kg/m^2^	25.0 ± 4.2	25.8 ± 4.3	23.7 ± 3.2	26.5 ± 5.9	6.2	0.002	b < a,c
Medication for physical illness, yes	24 (14.7)	11 (15.9)	9 (12.5)	4 (18.2)	0.7	0.683	
Smoker-current, yes	56 (34.4)	19 (27.5)	25 (34.7)	12 (54.5)	5.4	0.063	
Alcohol-current, yes	76 (46.6)	32 (46.4)	34 (47.2)	10 (45.5)	0.0	> 0.999	
Family history of mood disorders, yes	62 (38.0)	22 (31.9)	30 (41.7)	10 (45.5)	2.0	0.375	
Diagnosis					28.0	< 0.001	
Bipolar I disorder	72 (44.2)	47 (68.1)	20 (27.8)	5 (22.7)			a > b,c
Bipolar II disorder	91 (55.8)	22 (31.9)	52 (72.2)	17 (77.3)			a < b,c
Current status of mood episode					164.1	< 0.001	
Euthymia	69 (42.3)	69 (100.0)	0 (0.0)	0 (0.0)			a > b,c
(Hypo)mania	20 (12.3)	0 (0.0)	14 (19.4)	6 (27.3)			a < b,c
Depression	74 (45.4)	0 (0.0)	58 (80.6)	16 (72.7)			a < b,c
Age at bipolar disorder diagnosis, year	21.2 ± 8.1	23.2 ± 9.0	19.3 ± 6.7	21.1 ± 8.4	4.1	0.018	a > b
Frequency of hospitalization	1.6 ± 1.9	2.1 ± 2.0	1.2 ± 1.8	1.2 ± 1.3	4.6	0.011	a > b
Age at medication onset, year	24.4 ± 8.9	25.5 ± 10.2	23.2 ± 7.2	25.0 ± 9.3	1.2	0.307	
Lithium, yes	48 (29.4)	17 (24.6)	19 (26.4)	12 (54.5)	7.763	0.021	a, b < c
Valproate, yes	127 (77.9)	55 (79.7)	56 (77.8)	16 (72.7)	0.6	0.783	
Lamotrigine, yes	27 (16.6)	13 (18.8)	10 (13.9)	4 (18.2)	0.8	0.738	
Antipsychotics^††^, yes	135 (82.8)	50 (72.5)	64 (88.9)	21 (95.5)	8.9	0.010	a < b,c

(a) Stable-VPAR: Patients in remission state (not in depression or (hypo)mania) with an Alda A score of valproate ≥ 5

(b) Acute-VPAR: Patients in acute mood state (depression or (hypo)mania) with an Alda A score of valproate ≥ 5

(c) Acute-VPANR: Patients in acute mood state (depression or (hypo)mania) and an Alda A score of valproate < 5

BMI, body mass index; VPAR, Valproate responder; VPANR, Valproate non-responder

^†^chi-square test was performed for categorical variables, whereas analysis of variance with multiple comparisons (Tukey's HSD) was performed for continuous variables

^††^Antipsychotics includes aripiprazole, olanzapine, risperidone, and quetiapine

**Fig. 2:**
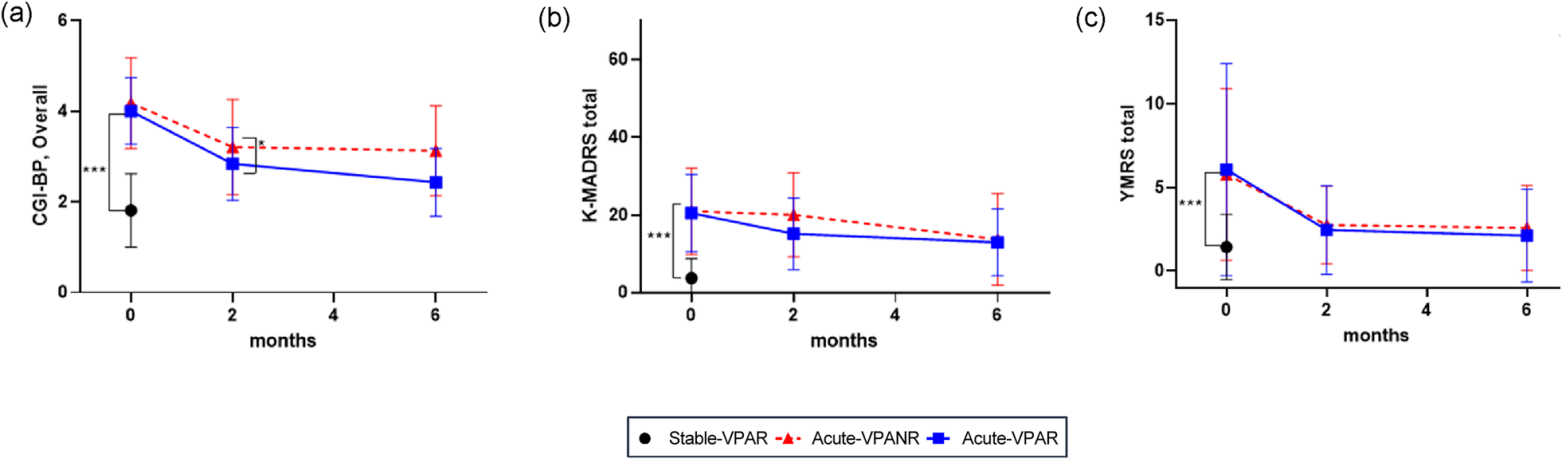
Comparison of changes in symptom severity between the three groups during different follow-up periods. This figure shows the comparison of changes in symptom severity between three groups (Stable-VPAR, acute-VPAR, and acute-VPANR) during different follow-up periods (T0: Baseline, T1: 8 ± 2 weeks, and T2: 6 ± 1 month) using **a** CGI-BP, overall, **b** K-MADRS, and **c** YMRS. VPAR, Valproate responder (Alda A score of Valproate ≥ 5); VPANR, Valproate non-responder (Alda A score of Valproate < 5); CGI-BP, Clinical global impression scale-bipolar disorder; K-MADRS, Montgomery–Asberg Depression Rating Scale; YMRS, Young mania rating scale. ***p < 0.001, ** *p* < 0.01, * *p* < 0.05

At T0, the acute groups exhibited significantly higher levels of overall BD symptoms (CGI-BP, Overall, K-MADRS, YMRS) than the stable-VPAR group (Fig. 2a–c). At T1, the acute-VPAR group showed a significantly lower overall severity (CGI-BP) than the acute-VPANR group (Fig. 2a). However, there were no differences in depression or manic symptoms between the acute-VPAR and acute-VPANR groups at T1 or T2 (Fig. 2b, c).

### Peptides that exhibited a distinct alteration in expression over time

**Fig. 3:**
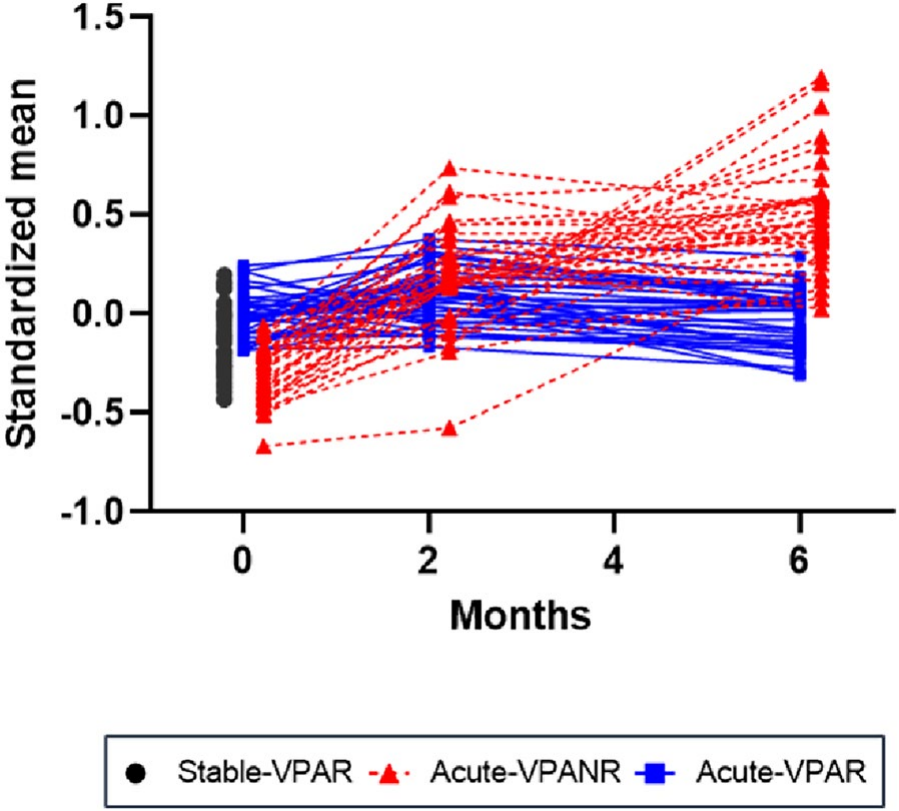
A plot of 37 significant peptides (from 27 proteins†) after linear mixed-effect model analysis. † CERU, Ceruloplasmin; FA12, Coagulation factor XII; A1AT, Alpha-1-antitrypsin; ANGT, Angiotensinogen; A2MG, Alpha-2-macroglobulin; APOA1, Apolipoprotein A-I; CRP, C-reactive protein; SAMP, Serum amyloid P-component; CO9, complement C9; VTDB, Vitamin D-binding protein; HEMO, Hemopexin; KLKB1, kallikrein B1; VTNC, Vitronectin; VWF, von Willebrand factor; PROS, Vitamin K-dependent protein S; CBG, Corticosteroid-binding globulin; FA5, Coagulation factor V; VINC, Vinculin; TAGL2, Transgelin-2; BTD, Biotinidase; RAP1B, Ras-related protein Rap-1b; 1433Z, 14-3-3 protein zeta/delta, tyrosine 3-monooxygenase/tryptophan 5-monooxygenase activation protein zeta; HGFA, Hepatocyte growth factor activator; SPP24, Secreted phosphoprotein 24; ITIH4, Inter-alpha-trypsin inhibitor heavy chain H4; FETUB, Fetuin B; TLN1, Talin-1; VPAR, Valproate responder (Alda A score of Valproate ≥ 5); VPANR, Valproate non-responder (Alda A score of Valproate < 5)

Of the 158 peptides, 37 (corresponding to 27 proteins) exhibited a significant interaction effect between group and time, as shown in Fig. 3 and Supplementary Table S3-1. As shown in Fig. 3, these 37 peptides exhibited a distinct pattern of increasing changes over the 6-month follow-up period in the acute-VPANR group compared with the pattern observed in the acute-VPAR group. Even after accounting for the influence of various covariates, such as age, sex, BMI, type of BD, and mood state (Supplementary Table S3-2–3–6), the observed statistical differences remained significant. Additionally, of the 37 peptides, 8 peptides (FETUB, A1AT, ITIH4, FA5, VWF, HGFA, BTD, VTNC) were also significant in the independent validation dataset (Supplementary Table S3-7).

### Peptides that exhibited differences regardless of overtime

Among the proteins examined in the three groups, 10 were differentially expressed. However, none of the proteins showed time-independent differential expression, which could reflect the response to valproate treatment regardless of the time factor both in the discovery and validation datasets, as shown in Supplementary Table S4-1 and S4-2.

### Candidate gene association studies

Twenty-seven candidate genes were selected from proteins that showed significant associations with response to valproate treatment in the proteomic study (Supplementary Table S5-1). A total of 1627 SNVs from 27 genes were included in the analyses. A total of 37 SNVs in 22 genes showed nominally significant associations with the Alda A continuous score (Supplementary Table S5-2), whereas 24 SNVs in 15 genes showed nominally significant associations (raw p < 0.05) with the acute-VPAR group (Supplementary Table S4-3). Among these SNVs, three (rs115788299, rs11563197, and rs117669164) in the secreted phosphoprotein 2 (SPP2) gene (encoding SPP24) reached gene-based FDR-corrected significance in the linear regression analysis with the Alda A continuous score. In particular, rs115788299 was a missense variant (T > A, C, G) (Asp > Glu). Of the common genetic variations that directly encoded proteins showing significant associations with valproate response (37 genes), 33 SNVs from 13 genes (CRP, TAGL2, FA5, ANGT, PROS, CERU, HGFA, KLKB1, CO9, 1433Z, VINC, vWF and VTNC) showed nominally significant associations with acute-VPAR group. In particular, 12 SNVs from 5 genes (FA5, HGFA, vWF and VTNC) encoding validated proteins showed significant associations with the acute-VPAR group (Supplementary Table 5–3).

### Biological pathway analysis

**Fig. 4:**
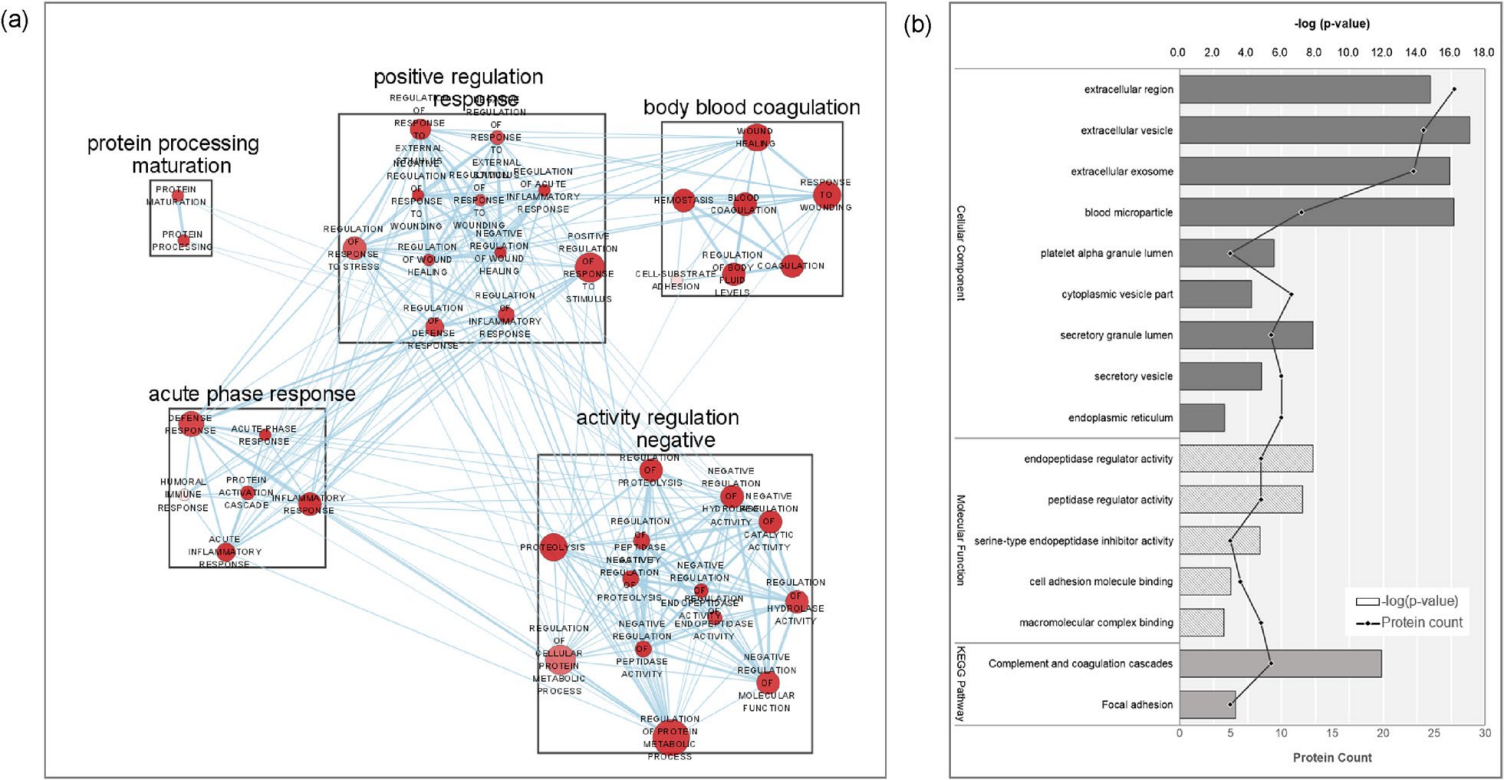
Bioinformatics analysis of proteins that reflected the response to Valproate treatment. **a** Network analysis showed the altered biological pathways that DEPs included; **b** GO enrichment map. Each node represents a GO term, and the connecting lines indicate common proteins shared between nodes. DEPs, Differentially expressed protein; GO, Gene ontology

In the bioinformatics analysis, we identified 27 proteins associated with response to valproate treatment. These proteins were associated with various biological processes, such as acute phase response, blood coagulation, protein processing maturation, positive regulation response, and negative regulation of activity (Fig. 4a). Analysis of the cellular components revealed that most protein markers were associated with the composition of the extracellular region (Fig. 4b). Analyses of molecular functions indicated that the markers were associated with activities such as endopeptidase regulation, binding to cell adhesion molecules, and binding to macromolecular complexes (Fig. 4b). The Kyoto Encyclopedia of Genes and Genomes pathway analyses revealed that the markers were associated with pathways involved in the complement and coagulation cascades and focal adhesions (Fig. 4b).

## Discussion

In the present study, we identified 27 protein markers significantly associated with response to valproate treatment, with 8 proteins (FETUB, A1AT, ITIH4, FA5, vWF, HGFA, BTD, VTNC) in the independent validation dataset also showing significance. Notably, 12 SNVs in the genes encoding FA5, HGFA, vWF, and VTNC showed significant associations, suggesting their validity as biomarkers of response to valproate treatment.

The majority of the significant proteins exhibited a progressive increase in expression over time in the acute-VPANR group (Fig. 3). The proteins that showed increasing pattern were associated with acute phase reaction (ceruloplasmin [CERU, *CP*], hemopexin [HEMO, *HPX*], and serum amyloid P-component [SAMP, *APCS*]), immune response (C-reactive protein [CRP, *CRP*], complement C9 [CO9, *C9*], and inter-alpha-trypsin inhibitor heavy chain H4 [ITIH4, *ITIH4*]), and coagulation cascades (alpha-2-macroglobulin [A2MG, *A2M*], coagulation factor V [FA5, *F5*], coagulation factor XII [FA12, *F12*], kallikrein B1 [KLKB1, KLKB1], von Willebrand factor [VWF, *VWF*]) (Fig. 4). These biological processes have been previously reported to be associated with psychiatric disorders, including BD, and can be suppressed by valproate treatment. (Ziani et al. 2022; Sluzewska et al. 1997; Maes et al. 1997; Kumar et al. 2019) These results indicate that valproate effectively inhibited the mechanisms associated with disease progression. This finding highlights the potential use of these protein markers as indicators for predicting and monitoring the efficacy of valproate treatment in patients.

Among the proteins examined in the genomic study, SNVs within the *SPP2* gene showed FDR-corrected significant associations. Notably, no prior study has reported the association of SPP2 with mental illnesses. However, our research team identified it as a target marker through a patient discovery set analysis. The SPP2 protein is mainly expressed in the liver and is primarily involved in mineral metabolism. Additionally, it serves as a barrier to prevent the spread of bone morphogenetic protein-2 (BMP-2), a bone-formation promoting protein with neurotoxic effect, to nervous system. (Tian et al. 2015) Thus, SPP2 might be associated with reduced response to valproate treatment through a neuroinflammatory process (Chen et al. 2018), although further studies are needed to confirm the association.

The present study successfully identified significant biomarkers indicative of the response to valproate treatment in patients with BD; however, this study had a few limitations. First, although the Alda score was used as the primary outcome measure, other measures to evaluate treatment responses did not align with the Alda score. The overall symptoms (CGI-BP) improved in the acute-VPAR group compared with those in the acute-VPANR group at the 2-month follow-up (T1), whereas there was no significant difference between the two groups at the 6-month follow-up (T2). The MADRS and YMRS scores showed no significant differences between the two groups at any time point. These clinical outcomes are believed to reflect the finding that the median duration of mood episodes in patients with BD is approximately 3 months rather than being solely indicative of the treatment response. (Solomon et al. 2010) The discordance between treatment response measures in our study might imply valproate could be used as an adjunctive agent for BD treatment. Moreover, although valproate has been suggested as a primary treatment agent for BD (Nierenberg et al. 2023), its efficacy is inferior to other first-line agents including lithium or atypical antipsychotics (Yildiz et al. 2023; Nestsiarovich et al. 2022). In fact, most patients in our study were under polypharmacy, which is very common in the real-world treatment of BD (Merikangas et al. 2011; Baek et al. 2014). Second, although the Alda Scale is designed for assessing treatment responses over a one-year duration, our study had applied it over a 6-month period. However, this time-period was considered suitable for evaluating early response to valproate in the acute phase of symptoms. In addition, we specifically utilized the Alda A score, focusing on the medication response evaluation while excluding the duration aspect. Third, we could not include stable VPANR in the analysis due to the limited number of participants. However, given that ineffective medications are typically recommended for discontinuation in the course of treatment (Qureshi and Young 2021), it is not uncommon for a small subset of patients undergoing long-term valproate treatment to be categorized as non-responders. Fourth, the inclusion of other medications taken by participants take may introduce bias into the results. This inclusion was unavoidable considering that most patients were under polypharmacy. However, we applied a statistical correction to account for potential confounding factors that might influence protein expression, including age, sex, BMI, and type of BD. Finally, although the FDR was considered, we could not completely correct for multiple comparisons.

In conclusion, our study identified significant proteomic and genomic markers that could serve as valuable indicators of the underlying mechanisms associated with the response to valproate treatment. These markers could help develop more effective therapeutic strategies for patients with BD. Further investigations are necessary to elucidate the specific mechanistic underpinnings of these protein markers and validate their clinical relevance in diverse patient populations.

### Supplementary Information

Supplementary Material 1.

Supplementary Material 2.

Supplementary Material 3.

Supplementary Material 4.

Supplementary Material 5.

## Data Availability

Datasets presented in this study may be available from the corresponding authors upon reasonable requests.

## Abbreviations

acute-VPAR: Acute-valproate responder
BD: Bipolar disorder
CGI-BP: Clinical Global Impression-Bipolar Version
DSM-5: Diagnostic and Statistical Manual of Mental Disorders 5
FDR: False discovery rate
SNVs: Single nucleotide variants
MS: Mass spectrometry
MRM-MS: Multiple reaction monitoring
YMRS: Young Mania Rating Scale
